# Association of Racial/Ethnic Segregation With Treatment Capacity for Opioid Use Disorder in Counties in the United States

**DOI:** 10.1001/jamanetworkopen.2020.3711

**Published:** 2020-04-22

**Authors:** William C. Goedel, Aaron Shapiro, Magdalena Cerdá, Jennifer W. Tsai, Scott E. Hadland, Brandon D. L. Marshall

**Affiliations:** 1 Department of Epidemiology, Brown University School of Public Health, Providence, Rhode Island; 2 Department of Medicine, Albert Einstein College of Medicine, Montefiore Medical Center, Bronx, New York; 3 Department of Population Health, New York University School of Medicine, New York; 4 Department of Emergency Medicine, Yale School of Medicine, Yale University, New Haven, Connecticut; 5 Department of Pediatrics, Boston University School of Medicine, Boston, Massachusetts; 6 Grayken Center for Addiction Medicine, Boston Medical Center, Boston, Massachusetts

## Abstract

**Importance:**

Treatment with methadone or buprenorphine is the current standard of care for opioid use disorder. Given the paucity of research identifying which patients will respond best to which medication, both medications should be accessible to all patients so that patients can determine which works best for them. However, given differences in the historical contexts of their initial implementation, access to each of these medications may vary along racial/ethnic lines.

**Objective:**

To examine the extent to which capacity to provide methadone and buprenorphine vary with measures of racial/ethnic segregation.

**Design, Setting, and Participants:**

This cross-sectional study included all counties and county-equivalent divisions in the US in 2016. Data on racial/ethnic population distribution were derived from the American Community Survey, and data on locations of facilities providing methadone and buprenorphine were obtained from Substance Abuse and Mental Health Services Administration databases. Data were analyzed from August 22, 2018, to September 11, 2019.

**Exposures:**

Two county-level measures of racial/ethnic segregation, including dissimilarity (representing the proportion of African American or Hispanic/Latino residents who would need to move census tracts to achieve a uniform spatial distribution of the population by race/ethnicity) and interaction (representing the probability that an African American or Hispanic/Latino resident will interact with a white resident and vice versa, assuming random mixing across census tracts).

**Main Outcomes and Measures:**

County-level capacity to provide methadone or buprenorphine, defined as the number of facilities providing a medication per 100 000 population.

**Results:**

Among 3142 US counties, there were 1698 facilities providing methadone (0.6 facilities per 100 000 population) and 18 868 facilities providing buprenorphine (5.9 facilities per 100 000 population). Each 1% decrease in probability of interaction of an African American resident with a white resident was associated with 0.6 more facilities providing methadone per 100 000 population. Similarly, each 1% decrease in probability of interaction of a Hispanic/Latino resident with a white resident was associated with 0.3 more facilities providing methadone per 100 000 population. Each 1% decrease in the probability of interaction of a white resident with an African American resident was associated with 8.17 more facilities providing buprenorphine per 100 000 population. Similarly, each 1% decrease in the probability of interaction of a white resident with a Hispanic/Latino resident was associated with 1.61 more facilities providing buprenorphine per 100 000 population.

**Conclusions and Relevance:**

These findings suggest that the racial/ethnic composition of a community was associated with which medications residents would likely be able to access when seeking treatment for opioid use disorder. Reforms to existing regulations governing the provisions of these medications are needed to ensure that both medications are equally accessible to all.

## Key Points

**Question:**

Does county-level capacity to provide methadone and buprenorphine vary with measures of racial/ethnic segregation?

**Findings:**

In this cross-sectional study of all 3142 counties or county-equivalent units in the US in 2016, counties with highly segregated African American and Hispanic/Latino communities had more facilities to provide methadone per capita, while counties with highly segregated white communities had more facilities to provide buprenorphine per capita.

**Meaning:**

These findings suggest that policy reforms are warranted to ensure equal access to both methadone and buprenorphine among all patients with opioid use disorder.

## Teaser

This cross-sectional study examines the associations of racial/ethnic segregation with county-level availability of methadone and buprenorphine in the US.

## Introduction

Since 1999, more than 700 000 people in the US have died of drug overdose, including more than 350 000 people who have died of opioid-involved overdose.^1,2,3^ Based on current trends, it is projected that 700 000 more individuals will die of opioid-involved overdose in the next decade.^4^ The current criterion standard for treatment of opioid use disorder (OUD), as approved by the Food and Drug Administration, includes methadone, buprenorphine, and naltrexone.^5^ In a 2018 retrospective cohort study in Massachusetts,^6^ all-cause and opioid-related mortality rates were decreased by half among adults who received methadone or buprenorphine within 1 year after a nonfatal opioid-involved overdose, underscoring the strong protective benefits of these medications. However, few individuals at risk for opioid-related harms receive these medications in a timely fashion.^7^

To date, there is little research identifying which patients with OUD will respond better to which medication.^8^ Furthermore, no single medication has been endorsed as a preferred first-line treatment,^5^ but a 2020 study^9^ reported that treatment with either methadone or buprenorphine was effective in reducing the risk of opioid overdose and opioid-related acute care utilization compared with other nonpharmacological treatment modalities. In addition, long-term outcomes do not appear to differ substantially between individuals using either medication.^10^ Given this lack of information to guide clinical practice, both medications should be equally available for all patients. However, treatment modalities for OUD are most often selected by patients on the basis of logistical considerations, such as geographic access to opioid treatment programs (OTPs) to provide methadone or health care practitioners with waivers to prescribe buprenorphine.^8^ Although both medications are approved by the Food and Drug Administration, there are stark differences in the social and historical contexts of their development, regulation, and initial implementation that continue to affect how these medications are delivered.

Methadone, a full μ-opioid receptor agonist, was shown to be effective in treating the symptoms of opioid withdrawal in the 1960s.^11,12,13^ Stemming from regulations issued when methadone was first approved by the Food and Drug Administration for the treatment of OUD in 1972,^14^ access remains tightly controlled. Outside its use as an analgesic, methadone can only be provided at OTPs that have been certified by the Substance Abuse and Mental Health Services Administration.^15^ As heroin use and crime became increasingly racialized in the media and political discourse the early 1970s with the beginning of the War on Drugs, OTPs were primarily targeted to urban African American communities with the goal of reducing crime.^16^ Furthermore, tight federal regulations governing operation of OTPs have created a punitive treatment environment for many patients, many of whom feel that they do not have control over when and where they are able to receive methadone, noting that the structure of these programs often controls their daily lifestyles.^17^ Given its origins in the War on Drugs, it is possible that racial/ethnic minority communities may have differential access to methadone compared with other evidence-based treatment options.

Buprenorphine, a partial μ-opioid agonist and κ-opioid receptor agonist, was first shown to be effective in treating opioid withdrawal in 1988.^18^ It was later approved for use in treating OUD in 2002.^18^ The Drug Addiction Treatment Act was passed in 2000 in an attempt to expand access, allowing health care practitioners with certain training to receive a waiver from the Drug Enforcement Administration to prescribe and administer scheduled drugs for use in treatment of OUD, including in office-based settings.^19^ As the first wave of the modern opioid epidemic escalated in predominantly white suburban and rural communities, there was a strong emphasis on expanding capacity to prescribe prescription buprenorphine in these settings.^20^ During congressional hearings on the Drug Addiction Treatment Act, buprenorphine was described by the director of the National Institute on Drug Abuse as a medication that would be useful for the new population of people who use opioids who were “unsuited to [methadone].”^20^ The availability of buprenorphine in office-based settings offered a new flexibility in treating OUD that was impossible within the highly regulated structure of OTPs.^21^ However, considering the framing of this medication at its introduction, it is possible that white communities may have differential access to buprenorphine.

It has been hypothesized that access to each of these medications may be racialized.^21^ Hansen and colleagues^22^ found that at a zip code level in New York, New York, the percentage of residents who were African American in an area was positively correlated with the proportion of residents who received methadone and negatively correlated with the proportion who received buprenorphine. The extent to which access to these medications for OUD may vary across racial lines at the national level remains unknown. As such, we conducted a cross-sectional, nationwide, geospatial assessment to examine the extent to which variation in capacity to provide methadone and buprenorphine at the county level was associated with residential racial segregation.

## Methods

This study was deemed to not be human participants research by the Brown University Human Research Protection Program and was considered exempt from institutional review board review and informed consent. The reporting of this cross-sectional study conforms to the Strengthening the Reporting of Observational Studies in Epidemiology (STROBE) reporting guideline.

### Primary Exposures

Two separate measures were created for each county to reflect the racial/ethnic distribution of the population (eTable 1 in the Supplement) using data from the American Community Survey. The first measure, the index of dissimilarity, represents the proportion of a group that would need to move to create a uniform distribution of the population by race/ethnicity.^23^ This index ranges from 0, indicating that the proportion of each group in each census tract is the same as the proportion in the population as a whole, to 1, indicating that each census tract contact contains only members of 1 group.^23^ Separate measures were calculated for African American residents and Hispanic/Latino residents. The second measure, the index of interaction, represents the probability that a member of one group will meet or interact with a member of another group.^23^ Its value can be interpreted as the number of people out of 100 that an African American person meets who will be white, and vice versa.^23^ This index ranges from 0 to 1 and, in general, is highest when both groups have equal numbers and are spread evenly across the tracts.^23^ Because this metric depends on both the distribution of groups and on the proportion of the population represented by each group, this index is not symmetrical (ie, the probability that a typical African American resident of a county will meet a white resident is not always the same as the probability that a typical white resident will meet an African American resident).^23^ As such, 4 separate measures were calculated for each county: 2 measures representing the interaction of African American residents and Hispanic/Latino residents with white residents and 2 measures representing the interaction of white residents with African American residents and Hispanic/Latino residents.

### Primary Outcomes

Data regarding the location of facilities providing methadone and buprenorphine in 2016 were obtained from the Substance Abuse and Mental Health Services Administration.^24,25^ For methadone, a facility was defined as a licensed OTP. For buprenorphine, a facility was defined as a location where at least 1 waivered health care practitioner was listed as maintaining a practice. Locations with multiple waivered practitioners were combined into a single listing. These facilities included licensed OTPs that also offered buprenorphine. As a sensitivity analysis, we removed facilities that offered both medications from our measures of capacity to provide buprenorphine so that a facility was considered to be a location where at least 1 waivered practitioner was listed as maintaining a practice but where methadone was not also being provided.

All postal addresses were preprocessed prior to geocoding to improve standardization and quality. Any addresses with post office boxes were removed. All data were reviewed for misspelled address information using Google Maps (Alphabet), and incorrect spellings were corrected. The spelling of addresses was standardized to US Postal Service format (eg, *street* to *St*). Addresses were then geocoded in ArcGIS version 10.4 (Esri) through a 2-step process to assign latitude and longitude coordinates to each facility.^26^ From these points, we created measures of capacity as the number of facilities per 100 000 population in a county.

### Other Covariates

#### Opioid-Involved Overdose Mortality

Using restricted use mortality files from the Centers for Disease Control and Prevention,^1,2,3^ we extracted data on the number of individuals who died of opioid overdose for all counties in the US and the District of Columbia.^27^ Opioid-involved mortality was included as a proxy for the underlying burden of OUD, which likely influences treatment capacity within and across counties. Opioid-involved overdose was expressed as the number of opioid-involved overdose deaths per 100 000 population in a county.

#### Urban/Rural Classification

The National Center for Health Statistics Urban-Rural Classification Scheme for Counties was developed for use in studying associations between urbanization level and health and for monitoring the health of urban and rural residents.^28^ Counties were classified into 6 urbanization levels on a continuum ranging from 1, indicating the most urban, to 6, the most rural. These categories were collapsed into 3 levels: metropolitan, micropolitan, and rural.

### Statistical Analysis

To assess the association of the primary exposures with capacity to provide methadone and buprenorphine, we computed separate ordinary least squares regression models for each racial/ethnic group and each medication. Spatial error models were computed as the residuals resulting from these models that showed evidence of spatial autocorrelation (ie, the degree to which a set of spatial features and their associated values tend to be clustered or dispersed in space),^29,30^ thus violating the assumption of uncorrelated errors terms in standard regression models.^31^ These models assume that the error terms across different spatial units are correlated with varying degrees based on how close the units are to each another.^31^ All final models were adjusted for opioid-involved overdose mortality and urbanicity. Four individual models were fit for each racial/ethnic group (African American and Hispanic/Latino) and each medication (methadone and buprenorphine). All analyses were conducted in R Studio statistical software version 1.1.456 (R Project for Statistical Computing). Data were analyzed from August 22, 2018, to September 11, 2019.

## Results

The analytic sample included 3142 counties. As of 2016, there were 1698 facilities providing methadone (0.56 facilities per 100 000 population) and 18 868 facilities providing buprenorphine (5.9 facilities per 100 000 population). The mean (SD) county-level capacity to provide methadone was 0.28 (0.96) facilities per 100 000 population, and the mean (SD) county-level capacity to provide buprenorphine was 4.1 (5.8) facilities per 100 000 population (Figure).

**Figure.**
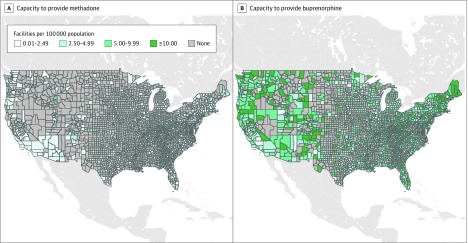
County-Level Capacities to Provide Methadone or Buprenorphine

The results of unadjusted and adjusted analyses of capacity to provide methadone are presented in Table 1. Capacity to provide methadone was lower in more dissimilar counties, where there were 0.42 fewer facilities per 100 000 population for every 1% increase in the proportion of African American residents who would need to move to create a uniform distribution of the population by race (β = −0.42; 95% CI, −0.67 to −0.18). Capacity to provide methadone was lower in counties where African American residents were more likely to interact with white residents, with 0.60 fewer facilities per 100 000 population for every 1% increase in the probability that an African American resident would interact with a white resident (β = −0.60; 95% CI, −0.83 to −0.37). However, the probability that a white resident would interact with an African American resident was not associated with capacity to provide methadone. Capacity to provide methadone was also lower in counties where white residents were more likely to interact with Hispanic/Latino residents, with 0.30 fewer facilities per 100 000 population for every 1% increase in the probability that a white resident would interact with a Hispanic/Latino resident (β =  −0.30; 95% CI, −0.50 to −0.08).

**Table 1.** Association of Racial Segregation With Capacity to Provide Methadone at the County Level in the US in 2016^a^

Community variable	β (95% CI)		
	Unadjusted	Adjusted	
		Model 1^b^	Model 2^c^
Index of dissimilarity			
African American	0.28 (0.09 to 0.47)	−0.42 (−0.67 to −0.18)	NA
Hispanic/Latino	0.58 (0.36 to 0.80)	NA	0.08 (−0.19 to 0.36)
Index of interaction			
White residents with African American residents	−0.99 (−1.73 to −0.26)	−0.79 (−1.58 to 0.00)	NA
African American residents with white residents	−0.75 (−0.92 to −0.58)	−0.60 (−0.83 to −0.37)	NA
White residents with Hispanic/Latino residents	−0.64 (−0.80 to −0.48)	NA	−0.30 (−0.51 to −0.08)
Hispanic/Latino residents with white residents	−0.71 (−1.20 to −0.21)	NA	0.06 (−0.46 to 0.58)
Urban/rural classification			
Metropolitan	[Reference]	[Reference]	[Reference]
Micropolitan	−0.15 (−0.25 to −0.06)	−0.09 (−0.19 to 0)	−0.10 (−0.19 to 0)
Rural	−0.35 (−0.43 to −0.28)	−0.18 (−0.27 to −0.10)	−0.20 (−0.29 to −0.11)
Opioid overdose deaths per 100 000 population	0.02 (0.01 to 0.02)	0.01 (0.01 to 0.02)	0.01 (0.01 to 0.01)

Abbreviation: NA, not applicable.

^a^ Capacity was measured in facilities per 100 000 population.

^b^ Includes urban/rural classification, opioid overdose deaths, and segregation measures for African American communities.

^c^ Includes urban/rural classification, opioid overdose deaths, and segregation measures for Hispanic/Latino communities.

The results of unadjusted and adjusted analyses of capacity to provide buprenorphine are presented in Table 2. Capacity to provide buprenorphine did not vary with the index of dissimilarity. However, capacity to provide buprenorphine was lower in counties where white residents were more likely to interact with African American residents, with 8.17 fewer facilities per 100 000 population for every 1% increase in the probability that a white resident would interact with an African American resident (β = −8.17; 95% CI, −12.70 to −3.63). This association was also observed in counties where white residents were more likely to interact with Hispanic/Latino residents, with 1.61 fewer facilities per 100 000 population for every 1% increase in the probability that a white resident would interact with a Hispanic/Latino resident (β = −1.61; 95% CI, −2.85 to −0.37). However, capacity to provide buprenorphine did not vary with the index of interaction for African American or Hispanic/Latino residents with white residents. These findings persisted in a sensitivity analysis in which OTPs providing buprenorphine were removed from the counts of facilities (eTable 2 in the Supplement).

**Table 2.** Association of Racial Segregation With Capacity to Provide Buprenorphine at the County Level in the US in 2016^a^

Community variable	β (95% CI)		
	Unadjusted	Adjusted	
		Model 1^b^	Model 2^c^
Index of dissimilarity			
African American	3.90 (2.78 to 5.02)	0.94 (−0.45 to 2.33)	NA
Hispanic/Latino	1.78 (0.52 to 3.05)	NA	−1.08 (−2.66 to 0.50)
Index of interaction			
White residents with African American residents	−13.12 (−17.49 to −8.74)	−8.17 (−12.70 to −3.63)	NA
African American residents with white residents	−3.26 (−4.26 to −2.25)	−1.00 (−2.33 to 0.32)	NA
White residents with Hispanic/Latino residents	−2.67 (−3.60 to −1.74)	NA	−1.61 (−2.85 to −0.37)
Hispanic/Latino residents with white residents	−3.62 (−6.46 to −0.78)	NA	−0.95 (−3.96 to 2.05)
Urban/rural classification			
Metropolitan	[Reference]	[Reference]	[Reference]
Micropolitan	−0.13 (−0.68 to 0.43)	0.29 (−0.24 to 0.82)	0.30 (−0.23 to 0.83)
Rural	−1.27 (−1.72 to −0.82)	−0.15 (−0.65 to 0.36)	−0.24 (−0.74 to 0.25)
Opioid overdose deaths per 100 000 population	0.12 (0.11 to 0.14)	0.12 (0.10 to 0.14)	0.10 (0.08 to 0.12)

Abbreviation: NA, not applicable.

^a^ Capacity was measured in facilities per 100 000 population.

^b^ Includes urban/rural classification, opioid overdose deaths, and segregation measures for African American communities.

^c^ Includes urban/rural classification, opioid overdose deaths, and segregation measures for Hispanic/Latino communities.

## Discussion

In this nationwide, population-based, cross-sectional geospatial analysis, we found substantial inequities in capacity to provide methadone and buprenorphine along racial lines in the US. Capacity to provide methadone was higher in counties where African American and Hispanic/Latino residents were unlikely to interact with white residents, while capacity to provide buprenorphine was higher in counties where white residents were unlikely to interact with African American or Hispanic/Latino residents. These findings describe a racialized treatment landscape for OUD in the US, where the racial/ethnic composition of a community may significantly determine whether residents will be able to access methadone rather than buprenorphine and vice versa when seeking treatment.

Much of the social and political attention surrounding the opioid epidemic has been centered on the dramatic increase in overdose deaths in nonurban, predominantly white communities.^32^ People who use drugs in these communities have been described by the news media as the “new face of addiction,”^32^ drawing a symbolic line between the drug-related harms experienced by these predominantly white communities and the harms experienced by urban African American and Hispanic/Latino communities since the 1970s. Although white adults have the highest absolute mortality rates due to opioid overdose as of 2017, the epidemic has accelerated rapidly among African American adults.^27^ Between 2009 and 2018, the age-adjusted rate of death due to opioid-involved overdose increased by 116% among white adults and by 289% among African American adults.^27^ Although capacity to provide methadone and buprenorphine is highest in counties with the highest disease burdens, the availability of methadone and buprenorphine is uneven across segregated counties, leaving many patients who do not respond to a medication without access to other evidence-based treatment options.

A 2016 study^33^ found racial differences in the modalities used to treat OUD. In analysis of patients receiving care through the Veterans Health Administration, African American patients were less likely to receive buprenorphine than their white counterparts. One explanation for the racial difference in use of these different treatment options may reflect differences in availability at the local level.^22^ This racialized pattern of enrollment in programs providing methadone was established during the period before buprenorphine was available,^34^ and as such, the characteristics of the patient population using methadone are thought to reflect the characteristics of individuals identified to have OUD during this period.^34^ However, these patterns have remained relatively unchanged since the expansion of access to buprenorphine.^35^ Our findings support this hypothesis, noting racially segregated patterns in capacity to provide methadone and buprenorphine at the national level.

Several strategies have the potential to improve capacity to provide to these medications. In the US, methadone can only be administered at certified OTPs.^15^ The number of OTPs has remained relatively stable since 2003.^36^ To meet treatment needs amid increasing burden of opioid-related harms, dispensing of methadone at a community pharmacy after a physician prescribes it in an outpatient clinic represents a viable solution.^37^ Despite the implementation of this approach in other countries,^37^ this approach is currently prohibited under federal law,^15^ but could be used to expand access to methadone. The administration of methadone at alternate locations could improve access in areas outside of the urban African American and Hispanic/Latino communities where these facilities are concentrated.^38^

Beginning in 2017, nurse practitioners and physician assistants in the US were able to complete the waiver process to administer buprenorphine under the Comprehensive Addiction and Recovery Act.^39^ Similar prescribing regulations in France resulted in increases in use of buprenorphine among people with OUD and large declines in opioid-involved overdose.^40^ Although policy initiatives like the Comprehensive Addiction and Recovery Act have been expected to increase access to buprenorphine,^41^ leading experts have sought to abolish this waiver process altogether.^42^ However, given large racial/ethnic disparities in access to care from physicians, physician assistants, and nurses,^43^ it is unlikely that deregulation alone would improve access to buprenorphine. Further efforts are needed to improve the availability of high-quality health services in these communities to ensure that both the medications and those able to provide to them are equally available to all who need them.

### Limitations

Our study has some limitations. First, our outcomes reflect 1 measure of potential capacity to provide methadone or buprenorphine, rather than true capacity and true access. Additional barriers to seeking and obtaining medications for OUD beyond availability of services at the county level are well documented.^44^ Furthermore, geographic access to a facility does not assure that a patient will subsequently receive evidence-based treatment. For example, facilities providing methadone that treat predominantly African American patients have been shown to be significantly more likely to dispense low doses inconsistent with best practices.^34,45^ In addition, our measure of technical capacity to provide buprenorphine does not account for practitioners with waivers who do not prescribe buprenorphine nor does it account for patient limit regulations that constrain practitioners to a maximum number of patients to whom they can prescribe.^19^ Second, our calculations reflect 1 measure of potential capacity to provide methadone or buprenorphine at the county level. As such, our findings may be different when conducted with finer geographic units.^46^ Third, our analyses do not incorporate measures of segregation for other racial/ethnic identities (eg, Native American/Alaskan Native, Asian, Native Hawaiian/Pacific Islander), and as such, our findings regarding access to these medications cannot be generalized to these populations.

## Conclusions

This cross-sectional study found that facilities providing methadone were significantly more likely to be located in highly segregated African American and Hispanic/Latino counties, while facilities providing buprenorphine were significantly more likely to be located in highly segregated white counties. The differential availability of medications for OUD across US counties represents an additional iteration of racism in the design and provision of health care services. Reforms to existing federal regulations governing the provisions of these medications are needed to ensure that both methadone and buprenorphine are equally accessible to all.

## Notes

Supplement. **eTable 1.** Formulas for Measures of Racial/Ethnic Segregation and Interaction at the County Level **eTable 2.** Association of Racial Segregation With Capacity to Provide Buprenorphine at the County Level, Excluding Opioid Treatment Programs Providing Buprenorphine
