# Supplementary material for: Association of Racial/Ethnic Segregation With Treatment Capacity for Opioid Use Disorder in Counties in the United States
Source: JAMA Netw Open. 2020 Apr 22;3(4):e203711. doi: 10.1001/jamanetworkopen.2020.3711 (PMC7177200; doi:10.1001/jamanetworkopen.2020.3711)
Supplement: Supplement. — eTable 1. Formulas for Measures of Racial/Ethnic Segregation and Interaction at the County Level eTable 2. Association of Racial Segregation With Capacity to Provide Buprenorphine at the County Level, Excluding Opioid Treatment Programs Providing Buprenorphine [file jamanetwopen-3-e203711-s001.pdf]

Supplementary Online Content

Goedel WC, Shapiro A, Cerdá M, Tsai JW, Hadland SE, Marshall BDL. Association of racial/ethnic segregation with treatment capacity for opioid use disorder in counties in the United States. *JAMA Netw Open*. 2020;3(4):e203711. doi:10.1001/jamanetworkopen.2020.3711

eTable 1. Formulas for Measures of Racial/Ethnic Segregation and Interaction at the County Level

eTable 2. Association of Racial Segregation With Capacity to Provide Buprenorphine at the County Level, Excluding Opioid Treatment Programs Providing Buprenorphine

This supplementary material has been provided by the authors to give readers additional information about their work.

© 2020 Goedel WC et al. *JAMA Network Open.*

eTable 1. Formulas for Measures of Racial/Ethnic Segregation and Interaction at the County Level

**Measure**	**Formula**	**Terms**
Dissimilarity	$\frac{1}{2}\sum_{i=1}^{n}\left\|\frac{w_i}{W_T}-\frac{b_i}{B_T}\right\|$	n = number of census tracts in county<br>w_i = number of white residents in tract *i*<br>W_T = number of white residents in county<br>b_i = number of Black/African American or Hispanic/Latinx residents in tract *i*<br>B_T = number of Black/African American or Hispanic/Latinx residents in county
Interaction	$\sum\left(\frac{n_{ib}}{N_b}\right)\left(\frac{n_{iw}}{n_i}\right)$	n_{ib} = number of Black/African American or Hispanic/Latinx residents in tract *i*<br>n_{iw} = number of white residents in tract *i*<br>N_b = number of Black/African American or Hispanic/Latinx residents in county<br>n_i = number of residents in county

© 2020 Goedel WC et al. *JAMA Network Open.*

eTable 2. Association of Racial Segregation With Capacity to Provide Buprenorphine at the County Level,[a] Excluding Opioid Treatment Programs Providing Buprenorphine

	**Unadjusted β (95% CI)**	**Adjusted (Model 1) β (95% CI)**	**Adjusted (Model 2) β (95% CI)**
**Index of Dissimilarity**			
Black/African American	3.74 (2.64, 4.84)	2.90 (2.12, 3.69)	----
Hispanic/Latinx	1.61 (0.36, 2.86)	----	-1.15 (-2.71, 0.42)
**Index of Interaction**			
White residents with African American residents	-12.87 (-17.19, -8.55)	-.814 (-12.62, -3.65)	----
African American residents with White residents	-3.00 (-4.00, -2.01)	-0.86 (-2.17, 0.45)	----
White residents with Hispanic/Latinx residents	-2.50 (-3.42, -1.58)	----	-1.55 (-2.78, -0.33)
Hispanic/Latinx residents with White residents	-3.44 (-6.26, -0.63)	----	-1.00 (-3.98, 1.97)
**Urban/Rural Classification**			
Metropolitan counties	Referent	Referent	Referent
Micropolitan counties	-0.09 (-0.64, 0.45)	0.30 (-0.22, 0.83)	0.30 (-0.22, 0.83)
Rural counties	-1.16 (-1.61, -0.71)	-0.09 (-0.59, 0.41)	-0.19 (-0.68, 0.30)
**Opioid Overdose Death Rate (per 100,000 population)**	0.12 (0.10, 0.14)	0.12 (0.10, 0.13)	-0.10 (0.08, 0.11)

[a]Measured in facilities per 100,000 population.

© 2020 Goedel WC et al. *JAMA Network Open.*
